# Supplementary material for: Marine oxygen production and open water supported an active nitrogen cycle during the Marinoan Snowball Earth
Source: Nat Commun. 2017 Nov 6;8:1316. doi: 10.1038/s41467-017-01453-z (PMC5673069; doi:10.1038/s41467-017-01453-z)
Supplement: Supplementary file 1 — Supplementary Information [file 41467_2017_1453_MOESM1_ESM.pdf]

Supplementary Note 1

Constraining detrital influence and post-depositional alteration

Crucial to our interpretations is establishing that N, Fe, and trace element (TE) data are not explained by detrital influence or post-depositional alteration alone. Post-depositional conditions and geochemical indicators suggest that $\delta^{15}N$ values are primary (Supplementary Figures 1-2). Under anoxic depositional conditions, there is little to no isotopic fractionation as organic matter breaks down and N substitutes into clays [1–3]. It is possible that under oxic conditions, a small (1–3‰) positive shift occurs. On a larger scale, regional metamorphism occurred at $\sim$ 250 to 300 °C [4], and local mineralogy and structure indicate no major metamorphism in sample locations. While no studies have specifically quantified metamorphic temperatures at sample locations, close spatial association with quartz dislocation creep in basement rocks suggests temperatures of $\sim$ 325 °C, and previous work has suggested temperatures experienced by nearby basement are similar to conditions experienced by cover rocks [5]. In addition, linear correlation between Zr (incompatible, fluid-immobile) and Cs (incompatible, fluid-mobile) in all sections suggests post-depositional fluid alteration was minimal (Fig. 4).

Nitrogen retention in sedimentary rocks is resilient to greenschist facies metamorphism, with primary values unaffected at temperatures up to 350 °C [6, 7]. If N were volatilized and lost with progressive metamorphism, concentration and $\delta^{15}N$ would be negatively correlated, which is not observed (Supplementary Figure 1). As N is likely found primarily as NH_4^+ in clays in these units, it should be strongly bonded into clay mineral lattices rather than weaker bonds in organic matter [8]. In addition, if changes in N concentration were caused by changes in detrital input alone, the ratio of N to Rb (similar geochemically) should correlate with Al_2O_3, an indicator of detrital input. Such correlation is not observed (Supplementary Figure 2).

Supplementary Note 2

Possible hydrothermal influence

Given anoxic deep waters, it is possible that far-traveled hydrothermal input could affect elemental input at our sections. Indeed, SGS has a high Fe/Mn ratio, which might be expected from input

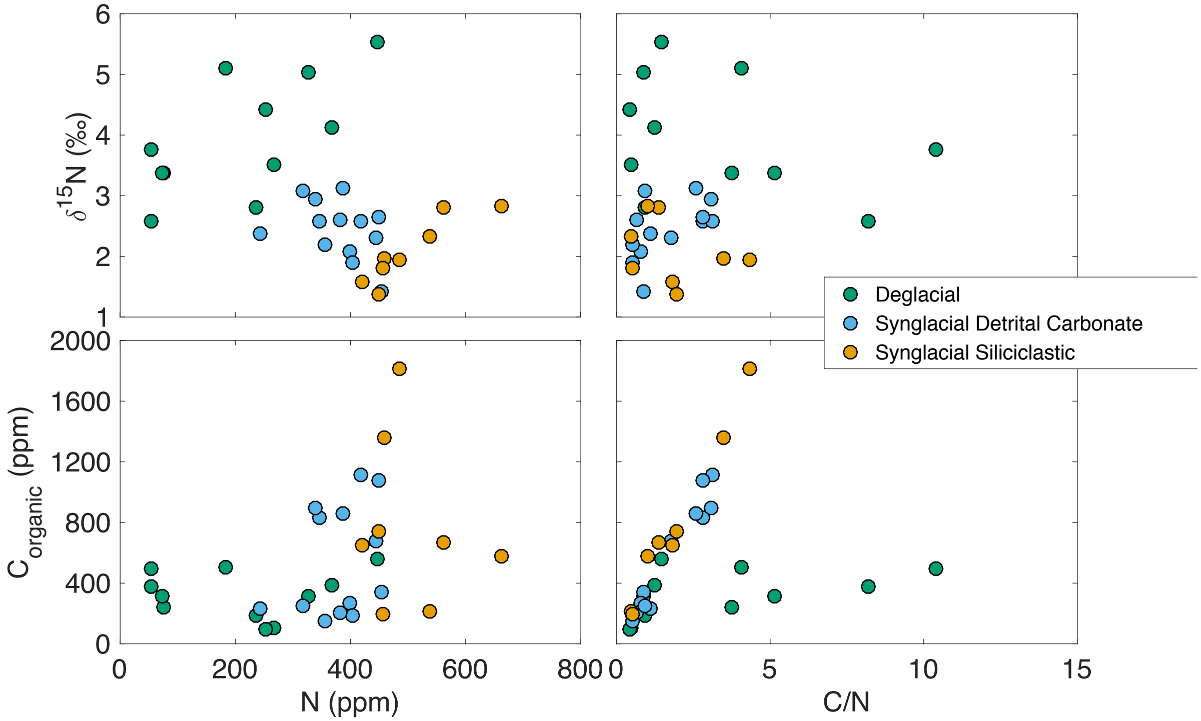

Supplementary Figure 1: **Nitrogen isotopes, C and N concentrations, and C/N ratio from decarbonated powders.** Lack of correlation between δ^{15}N and N concentration, as well as δ^{15}N and C/N, indicate the δ^{15}N values have not been altered during metamorphism. Carbon, however, seems to be altered, as strong correlation between C concentration and C/N indicates C loss. Additionally, there is no correlation seen between C and N concentrations, suggesting these two elements are decoupled in their preservation in measured sections.

and transport of soluble hydrothermal Fe (Supplementary Figure 5).

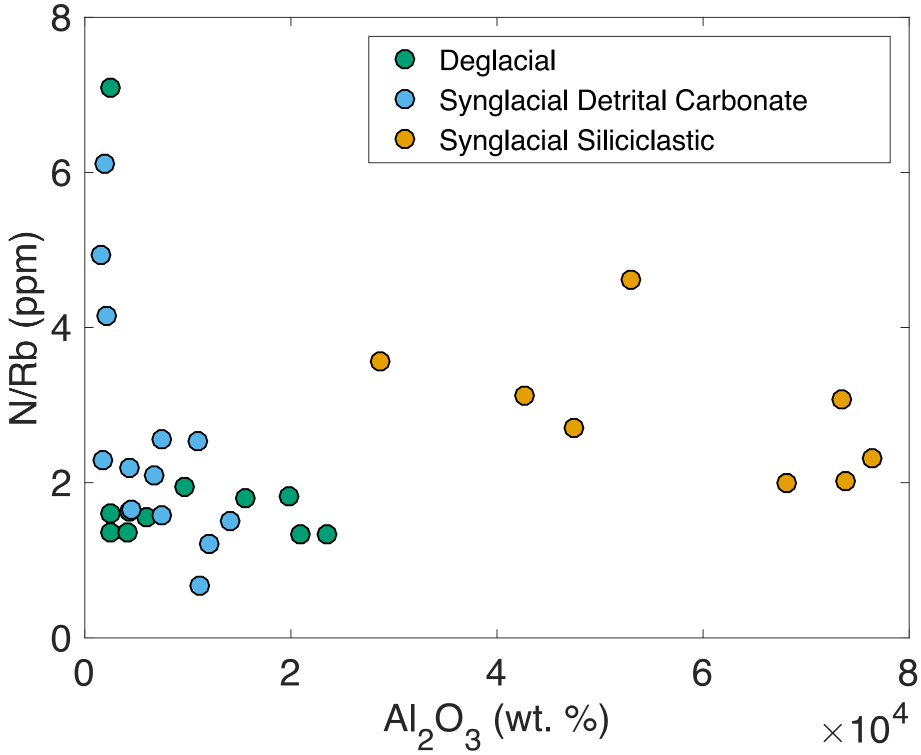

Supplementary Figure 2: **N/Rb vs Al2O3.** As with other trace elements, if changes in detrital input of N alone were responsible for variations in N concentration, we would expect a correlation between N/Rb and Al. There is no such correlation, corroborating little detrital influence.

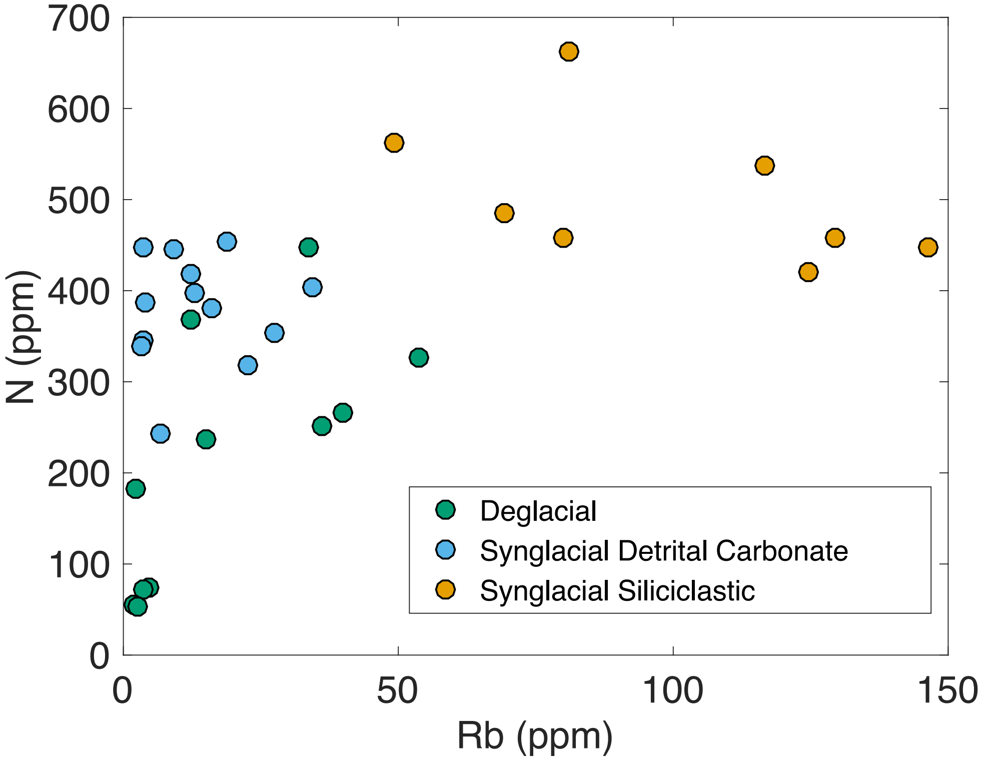

Supplementary Figure 3: **Nitrogen concentration plotted against Rb.** These species are similar in ionic size and charge when N is found as NH_4^+. The rough, positive correlation seen is consistent with N being contained primarily in clay minerals, where the majority of Rb is as well.

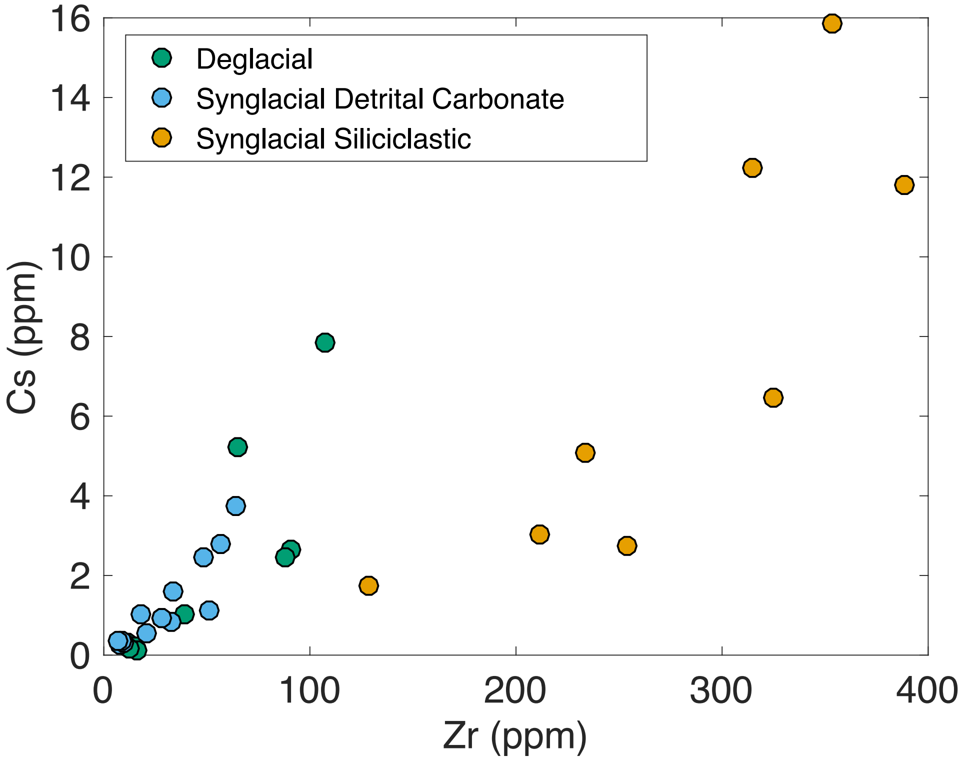

Supplementary Figure 4: **Caesium plotted against Zr whole rock analyses for all sections.** As Cs is fluid-mobile and Zr is not, if substantial fluid alteration had occurred in these units one would not expect a linear correlation between the two elements, since Cs would be preferentially removed. We suggest that these data are consistent with low fluid-alteration, and consistent with our interpretations of primary water-column chemistry and biologic productivity.

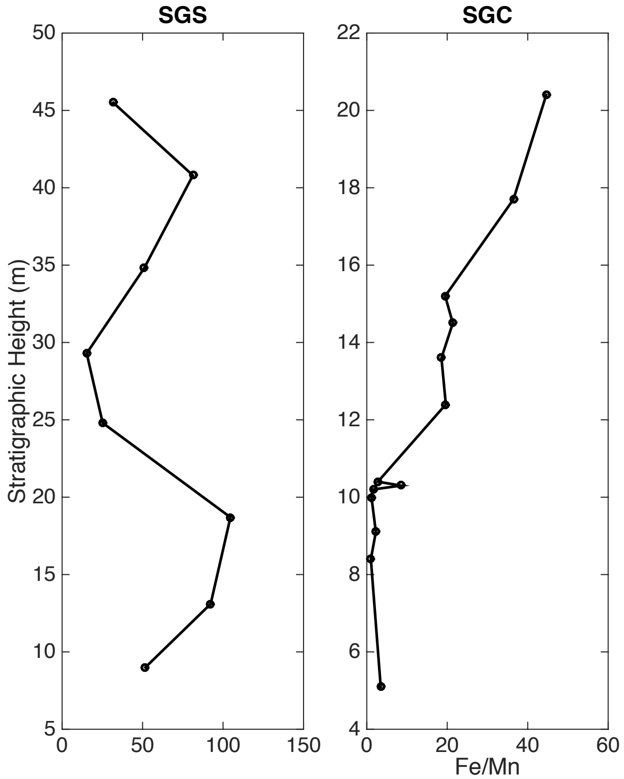

Supplementary Figure 5: **Fe/Mn ratio for three measured sections.** If there was prominent hydrothermal input into the system, high Fe/Mn ratios would be expected. While values are high in SGS, an anoxic deep ocean would tend to restrict the influence of hydrothermal input of Mo, V, and U as they would precipitate near a hydrothermal vent. We thus suggest that hydrothermal influence, at least in SGC and DGC are minimal.

Supplementary Table 1: Calculated element concentration from laser ablation analysis of standard NIST glass using BCR-2g and BIR-1a calibration based on [9]. Values for redox sensitive trace elements are within 5% of their accepted values with most others within 10%. Values for Na, Mg, Al, Si, K, Ti, Mn, and Fe are in weight % oxide, all others are in ppm.

**Element**	**Calculated**	**Accepted**
Na	14.6	12.87
Mg	0.08	0.07
Al	1.88	2.22
Si	70.5	69.6
P	0.11	0.12
K	0.054	0.06
Ca	12.1	11.71
Sc	507	455
Ti	0.075	0.08
V	441	450
Cr	529	408
Mn	0.003	0.05
Rb	405	417
Sr	530	492
Zr	520	437
Mo	416	417
Ba	432	430
La	462	426
Ce	452	447
Eu	496	444
Ho	545	440
Lu	533	430
U	448	452
Total Oxides	99.5	

Supplementary Table 2: Iron speciation and Fe/Al data, concentrations in weight %.

Sample	FeT	FeCarb	FeOx	FeMag	FePy	FeHR/FeT	FePy/FeHR	Fe/Al
J1413-45.5	5.22	0.11	0.91	0.19	0.003	0.23	0.002	0.29
J1413-40.8	4.49	0.12	0.71	0.11	0.004	0.21	0.004	0.46
J1413-34.8	4.32	0.09	0.63	0.04	0.005	0.18	0.007	0.62
J1413-29.3	3.25	0.63	0.82	0.04	0.006	0.46	0.004	0.54
J1413-24.8	2.57	0.26	0.93	0.12	0.003	0.51	0.002	0.76
J1413-18.7	4.22	0.38	0.73	0.06	0.003	0.28	0.003	0.59
J1413-13.1	3.51	0.44	1.08	0.13	0.005	0.47	0.003	0.85
J1413-9.0	2.17	0.71	0.78	0.03	0.003	0.70	0.002	1.83
J1409-7.8	2.36	0.14	1.06	0.08	0.004	0.54	0.003	0.51
J1409-7.1b	1.89	0.23	0.81	0.08	0.006	0.59	0.005	1.17
J1409-7.1a	0.22	0.19	0.02	0	0.006	0.98	0.028	1.60
J1409-6.3	0.35	0.33	0.02	0.001	0.002	1.00	0.006	4.26
J1409-4.6	0.10	0.10	0.01	0	0.003	1.06	0.028	2.96
J1409-3.6	0.31	0.27	0.01	0.01	0.004	0.95	0.014	0.71
J1409-2.6	0.75	0.66	0.08	0.01	0.004	1.02	0.005	0.10
J1409-1.6b	1.78	0.55	0.93	0.03	0.003	0.84	0.002	1.44
J1409-1.6a	0.39	0.38	0.03	0.001	0.003	1.06	0.007	0.11
J1409-0.6	3.52	0.43	1.97	0.07	0.004	0.70	0.002	0.90
J1409-0.1	1.20	0.40	0.69	0.03	0.004	0.93	0.004	3.94
J1408-20.4	0.39	0.21	0.09	0.002	0.003	0.78	0.010	3.24
J1408-17.7	0.16	0.06	0.02	0	0.003	0.53	0.036	6.23
J1408-15.2	0.36	0.19	0.13	0.01	0.002	0.90	0.006	4.57
J1408-14.5	0.13	0.05	0.04	0	0.003	0.70	0.033	5.73
J1408-13.6	0.16	0.11	0.04	0.003	0.003	0.98	0.019	5.87
J1408-12.4	0.76	0.32	0.37	0.003	0.004	0.92	0.006	1.66
J1408-10.2d	0.38	0.12	0.01	0	0.005	0.35	0.038	0.34
J1408-10.2c	2.00	0.95	0.05	0.02	0.004	0.51	0.004	3.48
J1408-10.2b	1.18	0.49	0.03	0.001	0	0.44	0.000	0.22
J1408-10.2a	0.94	0.44	0.03	0	0.01	0.51	0.021	0.09
J1408-9.1	1.06	0.50	0.02	0.03	0.004	0.52	0.007	0.79
J1408-8.4	1.09	0.48	0.03	0.02	0.003	0.49	0.006	0.21
J1408-5.1	1.42	0.60	0.09	0.01	0.004	0.50	0.006	0.35

Supplementary Table 3: Whole rock trace element data in ppm. Sample names are shown as Section-Stratigraphic height, where height is distance above the base of the formation. Samples from section J1408 are abbreviated “08”, those from J1409 “09”, and those from J1413 “13”. Samples with -A were run in duplicate, shown by -Dp. Several Decarbonate samples (Dc) were also run.

**Sample**	**Li**	**Be**	**B**	**Al**	**P**	**Sc**	**Ti**	**V**	^{52}**Cr**	^{53}**Cr**	**Mn**	**Co**	**Ni**	^{63}**Cu**	^{65}**Cu**	**Zn**	**Ga**	**Ge**
J1408-5.1	6.6	0.51	25	11000	459	3.1	714	26.3	14	14	1120	7.5	20	17	18	20	7.1	0.59
J1408-8.4	6.2	0.33	10	7560	478	2.2	443	11.4	7	6.8	1570	7.2	18	6.8	7.5	20	10	0.46
J1408-9.1	3.8	0.23	6.4	4600	364	1.6	278	6.89	4.9	4.8	1610	6.8	16	2.6	2.9	15	6.8	0.33
J1408-10.2c	16	0.5	33	14100	885	3.1	892	25.8	15	15	1120	6.9	27	4.9	5.5	17	7.1	0.77
J1408-10.2a	6.9	0.29	15	7380	513	2.2	449	13.5	7.3	7.1	1000	4.4	16	3.9	4.3	11	5.1	0.4
J1408-10.2d	1.5	0.13	5.2	2180	272	1.2	109	4.24	2.5	2.3	889	2.1	7.2	1.6	2	4.9	3.4	0.14
J1408-10.2b	11	0.36	22	11100	774	3.2	676	19.4	11	11	1370	7	25	7.1	8	17	10	0.54
J1408-12.4	20	0.45	23	12100	826	3.2	739	39.9	9.5	9.4	1020	10	53	36	38	23	12	0.61
J1408-13.6	2.3	0.09	4.5	2020	478	0.74	115	12.1	4.3	4.1	638	2	11	7.7	8.4	7.3	2.6	0.15
J1408-14.5	2.1	0.07	2.7	1640	318	0.68	87.5	5.75	2.5	2.3	438	1.7	9.7	6	6.6	5.4	2.7	0.13
J1408-15.2	5.3	0.27	11	6740	648	2.2	384	13.5	6	5.9	542	5.9	37	15	16	16	5.3	0.32
J1408-17.7	1.6	0.09	3.5	1760	375	0.6	97.8	3.56	2.2	2	300	1.5	9.8	16	17	3.8	1.4	0.15
J1408-20.4	4.9	0.2	11	4380	610	1.6	289	10.7	6.4	6.3	319	2.4	16	5.8	6.3	11	1.9	0.26
J1408-12.4-A	20	0.43	23	12100	807	3.3	731	40.1	9.3	9.2	1010	9.9	53	36	37	23	12	0.61
J1408-12.4-Dp	20	0.46	23	12000	845	3.2	747	39.7	9.6	9.6	1030	10	54	36	38	23	12	0.6
J1408-12.4-Dc	64	1.9	180	69300	9.87	7.2	5300	189	54	54	7.45	2.7	57	8.4	10	18	60	1.7
J1413-9.0	18	0.96	63	28600	1060	6.4	1760	60.1	39	38	1010	3.3	13	6.2	7.1	33	12	1.3
J1413-13.1	35	1.9	92	53000	804	12	3440	106	73	72	486	6.7	34	30	32	76	19	2.1
J1413-18.7	53	2.6	110	73500	766	19	4440	171	100	99	413	17	48	22	24	95	25	3
J1413-24.8	23	1.4	77	42700	1320	11	3050	86	61	60	1290	13	22	31	33	36	16	1.7
J1413-29.3	28	1.6	84	47400	1120	12	3280	96.8	67	66	1680	9.4	23	17	18	50	19	1.9
J1413-34.8	44	2.2	98	68100	984	17	4220	135	87	86	828	9.8	36	28	30	84	28	2.8

Continued on next page

Supplementary Table 3 – continued from previous page

Sample	Li	Be	B	Al	P	Sc	Ti	V	^{52}Cr	^{53}Cr	Mn	Co	Ni	^{63}Cu	^{65}Cu	Zn	Ga	Ge
J1413-40.8	51	2.7	110	76400	811	18	4490	149	93	92	432	19	40	21	23	120	30	3.4
J1413-45.5	51	2.5	110	73900	879	17	4510	146	91	89	681	13	40	51	55	91	30	3.2
J1413-45.5-A	51	2.5	110	73800	870	17	4500	146	90	89	669	13	40	52	55	92	30	3.3
J1413-45.5-Dp	51	2.5	110	74000	888	17	4530	145	91	90	693	13	40	51	54	91	30	3.2
J1413-45.5-Dc	37	3.1	200	75000	12.5	19	4780	144	78	78	9.69	0.9	2.9	1.1	2.3	18	34	2.4
J1409-0.1	4.8	0.45	11	6000	272	2.5	330	54.1	4.8	4.7	3990	16	73	39	41	26	2.7	0.67
J1409-0.6	23	1	40	21000	575	6.8	1100	261	14	14	5260	42	360	100	110	120	10	1.7
J1409-1.6b	20	0.88	33	19900	634	6.8	1150	74.3	16	16	5720	18	150	170	180	44	12	1.2
J1409-1.6a	1.6	0.23	4.5	2440	239	2.1	135	7.78	3.9	3.9	5570	13	62	22	23	30	1.3	0.32
J1409-2.6	5.5	0.36	15	9630	563	3.5	573	22.7	14	14	6490	20	110	32	33	30	6.4	0.57
J1409-3.6	0.75	0.21	5.4	4360	358	1.9	309	17.3	7	6.8	6650	22	80	150	160	22	2.4	0.29
J1409-4.6	1.4	0.17	3.7	2520	232	2	137	35.2	4.9	4.7	5900	20	62	7.8	8.5	27	37	0.15
J1409-6.3	1	0.18	4.4	4190	474	2.3	263	22.3	7.4	7.4	5230	19	53	4.5	4.9	28	6.2	0.27
J1409-7.1a	1.5	0.19	3.4	2460	272	2	157	20.2	3.9	3.7	6790	20	48	9.9	11	30	38	0.2
J1409-7.1b	9.7	0.84	27	15500	426	6	850	58.9	17	17	4910	15	64	19	20	39	8.1	1.3
J1409-7.8	18	1.2	47	23600	540	6.9	1410	65.2	17	17	3590	18	92	12	13	55	11	1.7
J1409-1.6b-A	21	0.85	33	19700	626	6.7	1150	73.7	16	15	5740	18	150	170	180	44	12	1.2
J1409-1.6b-Dp	20	0.91	32	20100	642	7	1140	75	16	16	5700	17	150	170	180	44	12	1.2
J1409-1.6b-Dc	63	2.1	130	67300	6.42	12	4450	109	52	51	28.6	1.8	130	1.4	2.5	30	37	2.1

Supplementary Table 4: WR trace element data continued

Sample	As	Rb	Sr	Y	Zr	Nb	Mo	Cd	Sn	Sb	Cs	Ba	La	Ce	Pr	Nd	Sm	Eu
J1408-5.1	20	19	700	13	51	2.3	0.12	0.14	0.53	0.1	1	190	9.6	18	2.7	11	2.5	0.67
J1408-8.4	0.24	13	700	8.9	33	1.4	0.27	0.09	0.29	0.1	0.8	320	7.6	14	2.2	9	2	0.47
J1408-9.1	0.16	6.7	600	7.2	21	0.87		0.07	0.19	0.04	0.5	220	6.2	11	1.8	7.7	1.6	0.37
J1408-10.2c	0.47	34	860	9.3	64	2.7		0.06	0.66	0.1	4	190	6.6	13	2.1	9.2	2.1	0.45
J1408-10.2a	0.44	16	1200	6.6	34	1.3		0.05	0.31	0.07	2	150	5	9.8	1.5	6.6	1.5	0.31
J1408-10.2d	0.28	3.7	1600	4	8.2	0.34	0.07	0.04	0.07	0.04	0.3	110	3.6	6.7	1.1	4.5	0.96	0.19
J1408-10.2b	0.95	23	1200	9.2	48	2.1	0.01	0.07	0.48	0.09	2	290	6.9	14	2.1	9.1	2.1	0.42
J1408-12.4	2.5	28	860	8.7	57	2.6	0.11	0.07	0.57	0.5	3	390	8.4	16	2.5	11	2.3	0.54
J1408-13.6	1.1	3.8	1000	3.6	9.3	0.38	0.35	0.06	0.07	0.04	0.3	76	1.7	2.8	0.61	2.8	0.64	0.14
J1408-14.5	1.2	3.4	850	3.3	8.6	0.3	0.04	0.05	0.06	0.04	0.3	85	1.8	3.4	0.62	2.8	0.62	0.15
J1408-15.2	1.2	12	1300	5.7	28	1.2		0.04	0.26	0.04	0.9	170	4.7	9.6	1.6	7	1.6	0.32
J1408-17.7	0.51	3.6	960	3.7	7.2	0.31	0.55	0.03	0.06	0.04	0.4	40	1.7	3.1	0.67	3.1	0.71	0.15
J1408-20.4	3.4	9.2	720	6.3	18	0.95	0.07	0.19	0.21	0.1	1	42	3.4	5.7	1.3	5.8	1.4	0.28
J1408-12.4-A	2.6	27	860	8.6	56	2.6	0.12	0.07	0.56	0.5	3	380	8.4	16	2.5	11	2.3	0.55
J1408-12.4-Dp	2.5	28	870	8.8	57	2.7	0.1	0.07	0.57	0.5	3	390	8.3	16	2.5	11	2.3	0.53
J1408-12.4-Dc	0.89	120	9.2	8.3	310	19	0.24	0.06	3.7	0.3	8	2000	0.18	2.4	0.09	0.68	0.52	0.24
J1413-9.0	1	49	340	14	130	5	0.73	0.12	1.2	0.4	2	300	11	21	2.8	12	2.7	0.88
J1413-13.1	5	81	160	21	250	9.5	2.2	0.2	2.5	2	3	470	21	39	5.1	20	4.3	0.89
J1413-18.7	2.6	120	78	28	320	12	0.34	0.13	3.5	0.7	6	590	33	60	7.6	30	6.1	1.2
J1413-24.8	2.7	69	400	35	210	8.4	1	0.18	1.8	0.7	3	370	28	53	7.3	31	7	1.4
J1413-29.3	9.3	80	370	28	230	8.9	0.76	0.14	2.1	0.4	5	470	27	51	6.9	28	6.2	1.3
J1413-34.8	25	120	150	25	310	12	0.56	0.13	3.1	0.5	10	720	36	66	8.2	32	6.4	1.2
J1413-40.8	4.1	130	51	28	390	15	0.24	0.14	3.5	0.9	10	800	33	59	7.8	31	6.3	1.2

Continued on next page

Supplementary Table 4 – continued from previous page

Sample	As	Rb	Sr	Y	Zr	Nb	Mo	Cd	Sn	Sb	Cs	Ba	La	Ce	Pr	Nd	Sm	Eu
J1413-45.5	2.5	150	74	27	350	13	1.6	0.18	3.4	0.7	20	790	27	50	6.5	26	5.4	1.1
J1413-45.5-A	2.5	150	74	27	360	13	1.4	0.17	3.4	0.7	20	790	28	51	6.6	26	5.5	1.1
J1413-45.5-Dp	2.6	140	74	27	350	13	1.7	0.19	3.4	0.7	20	790	27	50	6.4	26	5.4	1.1
J1413-45.5-Dc	0.43	110	27	25	480	19		0.09	4.3	0.4	4	930	3.5	7.4	0.93	3.9	1.1	0.32
J1409-0.1	4.3	12	77	11	28	1.4	1.1	0.16	0.27	0.3	0.9	77	5.6	11	1.8	8.1	2	0.45
J1409-0.6	9.5	40	72	18	91	3.8	1.5	1.1	0.9	1	3	270	11	22	3.4	15	3.5	0.73
J1409-1.6b	10	36	74	18	88	3.9	1.7	0.21	0.83	0.4	2	360	13	25	3.9	17	3.8	0.79
J1409-1.6a	0.58	4.8	100	11	12	0.43	0.01	0.16	0.12	0.04	0.3	36	3.9	8.2	1.5	7.2	1.8	0.44
J1409-2.6	0.76	15	88	13	39	1.9	0.3	0.22	0.42	0.07	1	210	8.9	15	2.5	11	2.6	0.62
J1409-3.6	0.42	3.8	93	9.5	15	0.95	1.5	0.19	0.19	0.05	0.2	68	7	11	1.9	8.2	1.6	0.34
J1409-4.6	0.45	1.7	110	8.1	12	0.39	0.2	0.2	0.1	0.07	0.2	1400	3.9	6.7	1.2	5.4	1.1	0.3
J1409-6.3	0.41	2.6	110	8.8	16	0.76	0.1	0.2	0.15	0.05	0.1	200	3.6	6.4	1.1	5	1.1	0.29
J1409-7.1a	0.6	2.4	130	8.4	12	0.47	0.04	0.22	0.11	0.06	0.2	1400	5.4	9.3	1.5	6.2	1.2	0.31
J1409-7.1b	13	34	68	11	65	2.2	0.65	0.23	0.6	0.3	5	210	8.4	15	2.2	9	2.1	0.51
J1409-7.8	34	54	71	13	110	5.2	0.34	0.2	1.1	0.5	8	280	15	26	3.7	15	3.1	0.62
J1409-1.6b-A	10	37	74	18	88	3.9	1.5	0.2	0.84	0.4	3	360	13	25	3.9	17	3.9	0.79
J1409-1.6b-Dp	11	36	74	18	88	3.9	1.8	0.21	0.82	0.4	2	350	13	25	3.9	17	3.8	0.78
J1409-1.6b-Dc	0.93	120	3.5	11	260	15	0.31	0.05	2.9	0.3	8	1100	0.48	2.3	0.15	0.77	0.45	0.15

Supplementary Table 5: WR trace element data continued

Sample	Gd	Tb	Dy	Ho	Er	Tm	Yb	Lu	Hf	Ta	W	Tl	^{206}Pb	^{207}Pb	^{208}Pb	Bi	Th	U
J1408-5.1	2.2	0.36	2.2	0.58	1.1	0.16	1	0.15	0.8	0.15	0.24	0.07	4.3	3.3	8.4	0.1	2	1.1

Continued on next page

Supplementary Table 5 – continued from previous page

Sample	Gd	Tb	Dy	Ho	Er	Tm	Yb	Lu	Hf	Ta	W	Tl	^{206}Pb	^{207}Pb	^{208}Pb	Bi	Th	U
J1408-8.4	1.7	0.28	1.6	0.43	0.81	0.12	0.77	0.11	0.53	0.1	0.23	0.05	5.9	4.6	12	0.1	1.3	0.86
J1408-9.1	1.4	0.23	1.3	0.36	0.66	0.1	0.62	0.09	0.34	0.06	0.14	0.03	2.1	1.6	4	0.06	0.96	0.62
J1408-10.2c	1.9	0.3	1.8	0.47	0.88	0.13	0.81	0.12	1.1	0.21	0.4	0.18	1.2	0.75	2	0.1	3	1.6
J1408-10.2a	1.3	0.21	1.2	0.32	0.6	0.09	0.56	0.08	0.56	0.1	0.19	0.08	1.5	1.1	2.8	0.06	1.4	0.95
J1408-10.2d	0.83	0.13	0.76	0.19	0.36	0.06	0.37	0.05	0.13	0.03	0.05	0.02	1.1	0.76	2	0.02	0.43	0.75
J1408-10.2b	1.8	0.3	1.7	0.45	0.84	0.12	0.76	0.11	0.82	0.14	0.3	0.11	2.4	1.7	4.5	0.1	2.1	1.1
J1408-12.4	2	0.31	1.8	0.47	0.87	0.13	0.82	0.12	1.1	0.2	0.47	0.24	6.9	5.1	13	0.3	2.6	2.4
J1408-13.6	0.6	0.1	0.59	0.16	0.3	0.04	0.26	0.04	0.14	0.03	0.05	0.03	1.1	0.72	1.8	0.02	0.37	0.93
J1408-14.5	0.58	0.09	0.56	0.15	0.28	0.04	0.26	0.04	0.14	0.02	0.07	0.04	0.86	0.58	1.5	0.01	0.41	0.72
J1408-15.2	1.3	0.21	1.2	0.3	0.55	0.08	0.49	0.07	0.49	0.09	0.17	0.14	2.1	1.5	3.7	0.05	1.4	1.3
J1408-17.7	0.63	0.1	0.6	0.16	0.3	0.04	0.27	0.04	0.1	0.02	0.06	0.06	0.55	0.3	0.77	0.01	0.38	0.88
J1408-20.4	1.2	0.19	1.1	0.28	0.53	0.07	0.46	0.07	0.31	0.07	0.15	0.07	1.6	1.2	2.9	0.03	0.97	0.93
J1408-12.4-A	2	0.31	1.8	0.46	0.87	0.13	0.84	0.12	1.1	0.19	0.48	0.24	7.1	5.3	13	0.3	2.6	2.4
J1408-12.4-Dp	2	0.31	1.8	0.47	0.87	0.13	0.8	0.12	1	0.2	0.45	0.24	6.7	5	13	0.2	2.6	2.4
J1408-12.4-Dc	0.82	0.2	1.7	0.55	1.2	0.22	1.5	0.22	5.7	1.6	2.1	1	0.97	0.33	0.83	0.2	7.1	7.6
J1413-9.0	2.5	0.43	2.6	0.69	1.3	0.21	1.3	0.19	2.2	0.39	0.69	0.27	3.3	2.5	6.6	0.05	4.7	2.5
J1413-13.1	4	0.68	4.2	1.2	2.3	0.35	2.3	0.35	4.6	0.75	1.3	0.52	9.6	7.3	19	0.3	8.1	3.9
J1413-18.7	5.5	0.92	5.7	1.6	3	0.48	3.1	0.47	6.1	0.99	1.7	0.64	8.6	6.5	17	0.4	12	3.5
J1413-24.8	6.7	1.2	7.2	1.9	3.5	0.52	3.3	0.47	3.7	0.61	1	0.43	8.2	6.2	16	0.2	7.2	3.5
J1413-29.3	5.7	0.96	5.6	1.5	2.8	0.43	2.8	0.41	4.2	0.68	1.1	0.41	7.3	5.5	14	0.2	8.3	3
J1413-34.8	5.5	0.87	5.1	1.4	2.7	0.42	2.7	0.41	5.8	0.97	1.4	0.73	5	3.7	9.7	0.2	12	3.3
J1413-40.8	5.7	0.94	5.8	1.6	3.1	0.49	3.2	0.5	6.7	1.1	2.3	0.71	11	8.7	23	0.4	13	3.3
J1413-45.5	5.1	0.88	5.5	1.5	3	0.47	3.1	0.48	6.5	1.1	1.5	0.86	8.8	6.7	18	0.6	11	4.2
J1413-45.5-A	5.1	0.88	5.5	1.5	3	0.47	3.1	0.48	6.6	1.1	1.5	0.87	8.8	6.7	17	0.6	11	4.2

Continued on next page

Supplementary Table 5 – continued from previous page

Sample	Gd	Tb	Dy	Ho	Er	Tm	Yb	Lu	Hf	Ta	W	Tl	^{206}Pb	^{207}Pb	^{208}Pb	Bi	Th	U
J1413-45.5-Dp	5.1	0.88	5.5	1.5	2.9	0.46	3.1	0.47	6.4	1.1	1.5	0.85	8.9	6.8	18	0.6	11	4.1
J1413-45.5-Dc	1.9	0.53	4.4	1.4	2.9	0.48	3.3	0.5	8.3	1.6	2.1	0.44	1.3	0.92	2.4	0.06	7.3	3.8
J1409-0.1	2	0.35	2.2	0.6	1.1	0.17	1.1	0.16	0.49	0.1	0.71	0.16	3	2.3	5.9	0.2	1	1.2
J1409-0.6	3.4	0.58	3.6	1	2	0.31	2.1	0.32	1.5	0.26	3	0.33	27	22	56	0.5	3.9	2.3
J1409-1.6b	3.5	0.59	3.6	0.98	1.9	0.3	1.9	0.29	1.6	0.29	0.95	0.31	6	4.8	12	0.4	4	1.6
J1409-1.6a	1.8	0.31	2	0.54	1	0.16	1	0.15	0.2	0.03	0.12	0.03	3.1	2.5	6.3	0.03	0.51	0.6
J1409-2.6	2.5	0.43	2.6	0.69	1.4	0.21	1.4	0.22	0.71	0.13	0.39	0.13	1.7	1.3	3.3	0.01	1.3	1.1
J1409-3.6	1.6	0.25	1.6	0.44	0.86	0.13	0.86	0.14	0.23	0.06	0.22	0.03	3.5	2.8	7.1	0.2	0.44	0.66
J1409-4.6	1.1	0.18	1.2	0.35	0.7	0.11	0.7	0.11	0.2	0.03	0.14	0.01	3.8	3.1	7.8	0.08	0.37	0.32
J1409-6.3	1.2	0.19	1.3	0.35	0.71	0.11	0.67	0.1	0.26	0.05	0.18	0.02	2.6	2	5.2	0.03	0.58	0.4
J1409-7.1a	1.2	0.2	1.2	0.36	0.74	0.11	0.76	0.12	0.2	0.03	0.13	0.02	2.7	2.1	5.3	0.03	0.5	0.48
J1409-7.1b	2.1	0.36	2.2	0.59	1.2	0.18	1.2	0.19	1.1	0.14	0.86	0.25	2.1	1.6	4.1	0.05	2.6	1.1
J1409-7.8	2.7	0.44	2.5	0.71	1.4	0.22	1.5	0.24	2	0.44	1.6	0.38	1.3	0.94	2.5	0.01	5	1.6
J1409-1.6b-A	3.5	0.59	3.6	1	2	0.3	1.9	0.29	1.6	0.29	0.96	0.32	6	4.8	12	0.4	4.1	1.6
J1409-1.6b-Dp	3.5	0.58	3.5	0.96	1.9	0.29	1.9	0.28	1.6	0.28	0.94	0.3	6	4.7	12	0.4	4	1.5
J1409-1.6b-Dc	0.73	0.2	1.7	0.58	1.4	0.27	2	0.33	4.7	1.2	2.8	0.94	0.51	0.29	0.78	0.2	4.1	3.5

Supplementary Table 6: Laser trace element data. Major elements (Na, Mg, Al, Si, P, K, Ca, Mn, and Fe) are given in wt. % X, all others in ppm. Sample names are shown as Section-Stratigraphic height-laser spot, where height is distance above the base of the formation and laser spot is defined as mineral-spot. As before, samples from section J1408 are abbreviated “08”, those from J1409 “09”, and those from J1413 “13”. For example, sample 08-5.1 4-1 means sample was collected 5.1 m above the base of the formation, and laser analysis took place on spot 1 in thin section mineral 4. Type indicates primary mineralogy: clay (C), carbonate (B), or quartz (Q). Clay spots were mostly clay, with some fine carbonate. Carbonate spots were usually larger carbonate grains with only minor clay, and quartz grains were distinct quartz crystals. There was also one oxide (X) spot measured.

**Sample**	**Type**	**Na**	**Mg**	**Al**	**Si**	**P**	**K**	**Ca**	**Sc**	**Ti**	**V**	**Cr**	**Mn**
J1408-5.1 4-1	Clay	0.17	11.9	6.9	15.5	0.8	2.6	59.3	6.4	0.33	102	52	0.02
J1408-5.1 4-2	Clay	0.69	30.9	1.7	8.1	0.1	0.4	51.5	3.5	0.03	28	15	0.03
J1408-5.1 4-3	Clay	0.08	17.5	4.7	21.1	0.1	3.6	43.9	5.6	0.21	123	47	0.02
J1408-5.1 5-2	Clay	0.39	13.3	6	26.2	0.3	2.2	56.1	6	0.32	126	72	0.02
J1408-5.1 5-3	Clay	0.51	8.9	7	25.9	0.3	2.7	57.9	7.3	0.46	105	77	0.01
J1408-5.1 5-4	Clay	0.12	18.3	5.4	16.6	0.2	2	53.4	5.6	0.53	84	59	0.02
J1408-5.1 3-4	Clay	0.05	15.9	1.9	4.5	0.1	0.7	73.9	4.4	0.08	27	21	0.02
J1408-10.2 1-1	Clay	0.36	23.2	9.7	28.7	0.4	4	30	8.5	0.68	107	105	0.02
J1408-10.2 1-2	Carb	0.08	2	1.2	3.3	0.1	0.6	93.3	3.3	0.03	20	15	0.01
J1408-10.2 1-3	Qtz	0.04	0.3	0	99	0	0	0	5.4		1	6	
J1408-10.2 1-4	Clay	0.09	25.1	5	16.2	0.2	2.4	48.1	5.2	0.26	49	56	0.02
J1408-10.2 1-5	Carb	0.12	1.2	0.1	1.5	0.1	0.1	98.2	1.2		3	1	0.01
J1408-10.2 2-1	Clay	0.12	19.7	2.6	8	0.4	1.8	67.8	2.9	0.17	33	19	0.01
J1408-10.2 2-2	Carb	0.5	0.8	0.1	8.1	0.1	0.1	91.4	0.7	0.01	2	8	
J1408-10.2 2-3	Clay	0.83	0.2	15.6	63.8	0.1	15.9	2.8	2.3		2	3	
J1408-10.2 2-4	Clay	0.11	29.1	6.9	20.7	1.3	3.7	37	3.6	0.59	85	59	0.02
J1408-10.2 2-5	Carb	0.14	2.1	0.1	15.3	0	0	83.2	1.5	0.02	3	4	0.01
J1408-10.2 3-1	Carb	0.08	0.3	0	1.4	0.1	0	99.5	0.4		1	0	
J1408-10.2 3-2	Carb	0.07	1.1	0.6	2.5	0.1	0.5	96.2	2.2		6		0.01
J1408-10.2 4-1	Qtz	0.66	0.8	0.1	96.1	0	0	1.8	3.5		2		
J1408-10.2 4-2	Carb	0.1	2	0.2	3.8	0	0.2	94.6	4		2	5	0.01
J1408-10.2 4-3	Carb	0.14	0.5	0	5.4	0.1	0	95	4.6		1	3	0.01
J1408-10.2 4-4	Clay	0.28	30.1	1.3	7.4	0.1	0.6	55.4	4.3	0.08	34	19	0.02
J1408-10.2 4-5	Clay	0.08	1.4	27.4	47.5	0.1	1.6	21.5	4.1	0.09	78	57	
J1408-10.2 4-6	Clay	0.67	20.4	1.9	9.4	0.1	0.6	66.4	4.5	0.32	20	19	0.02
J1409-1.6 1-1	Qtz	0.04	0.2	0	99.1	0		0	3.4		1	1	
J1409-1.6 1-2	Carb	0.16	37.7	0.1	1.3	0.1	0	62.1	1.2		1	6	
J1409-1.6 1-3	Clay	0.11	36.2	1.2	3.7	0.1	0.5	57.6	6.1	0.02	11	10	0.11
J1409-1.6 2-1	Carb	0.06	40.9	0	1.4	0.1	0	58.9	0.5		0	1	0.07

Continued on next page

Supplementary Table 6 – continued from previous page

Sample	Type	Na	Mg	Al	Si	P	K	Ca	Sc	Ti	V	Cr	Mn
J1409-1.6 2-2	Carb	0.21	44.4	0.1	1.4	0	0	55.5	0.3		0	2	
J1409-1.6 2-3	Clay	0.48	35.6	2.6	19.8	0.1	2.2	38.9	5.7	0.17	22	42	0.1
J1409-1.6 3-1	Qtz	0.03	0.1	0	99	0	0	0.1	4.2		2		
J1409-1.6 3-2	Carb	0.1	41.7	0	1.8	0.1	0.1	57.5	3.8		0		0.13
J1409-1.6 3-3	Clay	0.46	42.8	0	1.9	0.1	0	55.6	4.9		12	4	0.11
J1409-1.6 4-1	Qtz??	0.15	9.2	0.1	77.4	0	0.1	12.6	5.1		0	3	0.02
J1409-1.6 4-2	Clay	0.34	36.7	1.8	11.4	0.2	1.3	47.2	5.7	0.06	32	23	0.09
J1409-1.6 4-3	Clay	0.13	29.8	4.1	20.8	0.1	1.8	42.1	8.8	0.16	41	22	0.09
J1409-1.6 4-4	Oxide	0.54	10	1.8	43.7	0.3	1	13	4.2	2.41	245	19	0.03
J1409-1.6 5-1	Clay	0.11	26.8	7.7	28.3	0.1	3.2	32.4	14	0.53	75	46	0.06
J1409-1.6 5-2	Clay	0.12	24	4.5	34.5	0.3	2.8	32.1	11	0.43	97	54	0.08
J1409-1.6 5-3	Clay	0.34	14.4	12.4	50	0	2.1	18.4	8.2	0.1	119	19	0.06
J1409-1.6 6-1	Carb	0.06	39	0.6	12.3	0.1	0.1	47.9	4.9		8	3	0.07
J1409-1.6 6-2	Carb	0.5	38.3	0.8	5	0	0.4	54.3	8.6	0.03	15	7	0.11
J1413-24.8 2-1	Qtz	0.04	0	0.1	98.5	0	0	0.6	3.7	0.04	2	3	
J1413-24.8 2-2redo	Qtz	0.15	0	0.2	96.4	0	0.2	2.4	3.8		1	18	
J1413-24.8 2-3	Qtz	0.41	0	0	97.8	0	0	0.7	3.8	0.01	1	6	
J1413-24.8 2-4	Clay	2.46	7.3	12.8	42.2	0.1	3.3	17	19	1.23	136	128	
J1413-24.8 3-1	Qtz	0.44	0	0.1	98.3	0.1	0	0.4	5.8		2	6	
J1413-24.8 4-1	Qtz	0.05	0	0	99.1	0	0	0.2	3.5	0.01	1	4	
J1413-24.8 6-1	Clay	0.11	0.6	2.4	82.7		1	9.7	14	0.04	32	41	
J1413-24.8 6-2	Clay	0.75	22.9	2.2	6.1	0.1	1.7	49.7	5.1	0.07	24	12	0.01
J1413-24.8 6-3	Clay	0.09	17.1	2.4	11	0.1	0.1	43.5	9.6	0.04	91	30	0.01

Supplementary Table 7: LA data continued

Sample	Rb	Sr	Zr	Mo	Ba	La	Ce	Eu	Ho	Lu	U	Oxide sum
J1408-5.1 4-1	65	972	89.7	0.11	607	17	35	1.53	0.78	0.28	4.9	100.6
J1408-5.1 4-2	24	465	15.5	0.27	101	6.9	15	0.96	0.28	0.03	1.2	101.2
J1408-5.1 4-3	49	693	44.4	0.32	418	12.5	24	0.77	0.5	0.19	4.5	95.5
J1408-5.1 5-2	56	754	60.5	0.14	518	10.2	23	1.23	0.54	0.16	3.2	108.6
J1408-5.1 5-3	72	1040	248	0.19	583	15.4	32	1.79	1.2	0.45	10	106.1
J1408-5.1 5-4	52	678	68.1	0.23	492	9.8	20	1.02	0.48	0.21	4.3	101.3

Continued on next page

Sample	Rb	Sr	Zr	Mo	Ba	La	Ce	Eu	Ho	Lu	U	Oxide sum
J1408-5.1 3-4	23	1150	720	0.17	171	12.9	27	1.14	0.52	0.27	1.1	101.2
J1408-10.2 1-1	110	463	130	1.27	485	4	12	0.67	0.52	0.16	6	99.9
J1408-10.2 1-2	17	1530	6.1	0.22	82	16.5	35	0.86	0.61	0.23	1	101.3
J1408-10.2 1-3	0	0	0.2	0.16	1	0.1	0					99.5
J1408-10.2 1-4	68	747	41.8	0.34	288	6.8	19	0.61	0.42	0.14	2.3	100.7
J1408-10.2 1-5	0	4520	0.5		19	6.9	17	0.32	0.26	0.07	0.3	101.4
J1408-10.2 2-1	49	1400	53.6	6.16	156	6.2	16	0.59	0.38	0.16	3.6	101.1
J1408-10.2 2-2	3	1680	0.6	0.48	25	3.6	7	0.16	0.06	0.03	0.1	101.2
J1408-10.2 2-3	320	124	0.3	0.11	358	0.4	1	0	0.01	0.01	0.1	99.4
J1408-10.2 2-4	92	650	76.6	0.33	534	3	9	0.49	0.29	0.11	4.2	100.4
J1408-10.2 2-5	4	2420	2.1	0.07	105	5.5	10	0.33	0.17	0.07	0.6	101.1
J1408-10.2 3-1	0	1850	0.1		7	3.3	5	0.02	0.01	0	0.1	101.4
J1408-10.2 3-2	19	2560	3.1	5.59	108	5.3	10	0.53	0.52	0.21	0.8	101.4
J1408-10.2 4-1	0	31			8	0	1					99.5
J1408-10.2 4-2	3	1940	1.2		62	3.6	8	0.24	1.03	0.23	0.2	101.3
J1408-10.2 4-3	1	4480	2.7		13	11.5	30	1.92	1.57	0.49	0.2	101.3
J1408-10.2 4-4	15	972	18.9	0.11	90	6.3	19	0.98	0.36	0.05	1	101.2
J1408-10.2 4-5	45	316	12.9	0.06	153	3.6	6	0.08	0.1	0.01	0.3	100
J1408-10.2 4-6	16	1300	58.4	0.08	89	7.4	15	0.47	0.55	0.19	1.6	100.9
J1409-1.6 1-1	0	1	0	0.17	0	0.1	0			0.01		99.5
J1409-1.6 1-2	4	391	0.5	0.13	22	5.7	11	0.55	0.5	0.23	2.3	101.6
J1409-1.6 1-3	9	213	23.7	0.03	63	4.9	11	1.01	1	0.39	0.7	101.5
J1409-1.6 2-1	0	112	0.6	0.83	7	4.7	9	0.51	0.36	0.19	0.3	101.6
J1409-1.6 2-2	0	226	0.5	0.35	11	0.7	2	0.1	0.06	0.01	0.4	101.6
J1409-1.6 2-3	51	130	42.7		264	4.9	23	0.66	1.1	0.35	3.9	100.9
J1409-1.6 3-1	0	2	3.9		2	0	0			0	1.6	99.5
J1409-1.6 3-2	0	179	0.3		3	8	20	0.89	0.84	0.34	0.1	101.6
J1409-1.6 3-3	0	189	1.1		11	7.2	16	1.53	0.88	0.32	0.2	101.6
J1409-1.6 4-1	1	48	0.5	0.79	47	1.2	4	0.16	0.16	0.04		99.9
J1409-1.6 4-2	29	137	24.5		129	8	16	1.38	1.3	0.4	3.5	101.2
J1409-1.6 4-3	48	163	83.8	0.39	248	5.4	14	1.2	1.4	0.48	2.1	100.8
J1409-1.6 4-4	15	49	44.4	13.9	119	5.9	18	0.9	0.94	0.5	2.5	97.4
J1409-1.6 5-1	94	106	78.8	0.33	422	3.6	9	0.7	1	0.4	120	100.2
J1409-1.6 5-2	54	120	62.5		332	5.8	18	1.77	1.14	0.32	1.5	100.3
J1409-1.6 5-3	65	69	44	0.89	264	1.9	6	0.52	0.62	0.22	0.9	100.1

Continued on next page

Supplementary Table 7 – continued from previous page

Sample	Rb	Sr	Zr	Mo	Ba	La	Ce	Eu	Ho	Lu	U	Oxide sum
J1409-1.6 6-1	3	137	2.4		29	4	8	0.49	0.65	0.17	0.4	101.3
J1409-1.6 6-2	10	202	7.1	1.39	95	4.9	13	1.09	1.3	0.3	0.5	101.4
J1413-24.8 2-1	1	9	0.3	0.36	6	0.2	1	0.02	0.01		0.4	99.4
J1413-24.8 2-2redo	10	3	0.6		3		2		0.01		0.1	99.5
J1413-24.8 2-3	1	17	0.3		3	0.1	1				0.2	99.5
J1413-24.8 2-4	110	104	127	3.29	495	28.4	75	1.27	1	0.44	6.3	98.7
J1413-24.8 3-1	2	1	0.2	0.19	2	0.1	0			0.01	0.1	99.5
J1413-24.8 4-1	1	1	0.1		3	0.5	0	0.01			0	99.5
J1413-24.8 6-1	33	59	13.9	2.03	108	9.8	8	1.18	0.08	0.29	1.2	99.6
J1413-24.8 6-2	25	210	47.3	2.46	79	52.5	92	2.26	0.65	0.23	4.9	100.9
J1413-24.8 6-3	10	161	85.3	4.2	33	47.7	101	2.66	1.2	0.54	6.9	100.7

Supplementary Table 8: Nitrogen and C stable isotope analyses, performed at the University of Washington Isolab. As before, samples from section J1408 are abbreviated "08", those from J1409 "09", and those from J1413 "13". MR is the McRae shale, a UW internal standard. Sample mass is decarbonated sample powder mass analyzed. N (mg) and C (mg) are mass of each element analyzed by the mass spec, obtained by peak area integration, while TN and TC (ppm) are concentration of each element in decarbonated powder. Isotope values are also given along with atomic C/N ratio. Insoluble is the fraction of sample left after decarbonation, thus whole rock N (WR N) and whole rock C (WR C) are concentrations corrected for dissolved fraction in ppm. N- and C-peak areas from mass spectrometer analysis are given, along with blank area and N-peak/blank ratio. As these ratios are mostly above 10, the contribution of the blank to sample signal is minimal. Ages are given, and are based on relative stratigraphic position.

Sample	mass (mg)	N (mg)	TN	δ^{15}N	C (mg)	TC	δ^{13}C	C/N	Ins.	WR N	WR C	N-peak	C-peak	N-blank	Peak/blank	Age
13-13.1	61.41	0.0403	656	2.93	0.0351	572	-19.87	1.02	0.57	371	324	144.93	26.30	3.77	38.48	646.71
13-13.1	79.71	0.0533	669	2.72	0.0462	579	-19.60	1.01	0.57	379	328	197.74	34.95	2.79	70.87	646.71
13-29.3	55.05	0.0268	487	2.51	0.0753	1368	-16.74	3.28	0.47	230	646	96.48	56.43	3.77	25.62	645.85
13-29.3	79.98	0.0355	444	1.69	0.1084	1355	-16.60	3.56	0.47	210	640	130.83	81.57	3.34	39.20	645.85
13-29.3	108.98	0.0483	444	1.72	0.1479	1357	-16.56	3.57	0.47	210	641	177.83	111.42	3.30	53.84	645.85
08-5.1	56.25	0.0248	440	1.20	0.0182	323	-22.16	0.86	0.10	46	34	89.01	13.62	3.77	23.63	640
13-45.5	47.39	0.0212	447	1.20	0.0352	744	-17.71	1.94	0.66	295	490	76.14	26.41	3.77	20.22	645
09-7.8	52.50	0.0165	315	4.59	0.0056	107	-26.79	0.4	0.22	69	23	59.38	4.21	3.77	15.77	633.9
09-0.6	55.76	0.0156	279	3.46	0.0065	117	-18.37	0.49	0.20	56	24	56.00	4.87	3.77	14.87	634.9
13-34.8	86.18	0.0374	434	1.49	0.0577	670	-19.26	1.8	0.59	258	398	134.35	43.25	3.77	35.67	645.57
09-2.6	54.30	0.0129	237	2.80	0.0101	185	-24.67	0.91	0.12	29	22	46.33	7.54	3.77	12.30	634.6
13-24.8	59.16	0.0299	505	1.86	0.1078	1822	-16.26	4.2	0.45	225	811	107.52	80.74	3.77	28.55	646.18
09-6.3	81.48	0.0044	54	2.31	0.0318	390	-5.64	8.39	0.07	4	26	15.91	23.84	3.77	4.22	634.3
13-18.7	52.81	0.0290	549	2.24	0.0119	224	-22.22	0.48	0.67	367	150	104.25	8.88	3.77	27.68	646.42
09-1.6b	64.34	0.0161	250	4.25	0.0060	93	-21.47	0.43	0.26	66	25	57.95	4.47	3.77	15.39	634.8
09-0.1	57.56	0.0209	363	4.03	0.0221	384	-26.18	1.23	0.05	19	20	75.08	16.56	3.77	19.94	635
13-40.8	71.40	0.0326	456	1.99	0.0145	202	-22.59	0.52	0.65	299	132	117.08	10.83	3.77	31.09	645.29
09-1.6a	93.40	0.0067	72	3.00	0.0226	242	-15.56	3.93	0.10	7	25	24.14	16.96	3.77	6.41	634.7
13-9.0	64.82	0.0376	580	3.02	0.0441	680	-16.77	1.37	0.31	181	212	135.28	33.03	3.77	35.92	647
08-10.2c	77.46	0.0329	425	2.12	0.0148	191	-23.75	0.53	0.13	55	25	121.17	11.11	3.80	31.93	639.5
08-9.1	59.49	0.0142	239	2.03	0.0135	226	-23.25	1.1	0.05	11	10	52.33	10.08	3.80	13.79	639.67
08-10.2d	45.11	0.0156	345	2.57	0.0376	833	-26.01	2.81	0.04	15	37	57.32	28.14	3.80	15.10	639.17
08-15.2	60.11	0.0252	419	2.58	0.0670	1115	-27.10	3.1	0.06	26	68	92.74	50.22	3.80	24.44	638.33
08-8.4	75.27	0.0300	398	2.08	0.0200	266	-25.06	0.78	0.08	33	22	110.32	14.99	3.80	29.07	639.83
09-4.6	77.85	0.0043	55	3.77	0.0385	495	-18.60	10.4	0.04	2	21	15.89	28.84	3.80	4.19	634.4
09-7.1a	46.85	0.0059	126	4.40	0.0336	716	-17.75	6.62	0.05	6	35	21.76	25.15	3.80	5.73	634.2
08-14.5	28.03	0.0095	340	2.94	0.0250	892	-22.12	3.06	0.05	17	45	35.07	18.74	3.80	9.24	638.5
08-10.2b	61.42	0.0197	320	3.15	0.0157	255	-25.32	0.93	0.05	16	12	72.33	11.74	3.80	19.06	639
08-20.4	53.25	0.0235	441	2.29	0.0355	667	-22.93	1.76	0.05	20	30	86.40	26.60	3.80	22.77	638
08-17.7	36.79	0.0165	448	2.64	0.0397	1080	-22.36	2.81	0.02	8	20	60.71	29.76	3.80	16.00	638.17
09-7.1b	73.22	0.0175	239	5.82	0.0221	302	-21.38	1.47	0.14	33	41	64.40	16.57	3.80	16.97	634.1
08-13.6	43.73	0.0169	386	3.12	0.0377	861	-22.52	2.6	0.06	23	52	62.08	28.21	3.80	16.36	638.67
09-3.6	85.87	0.0061	72	3.33	0.0277	323	-8.52	5.27	0.08	6	27	22.60	20.79	3.80	5.96	634.5
08-10.2a	65.72	0.0250	381	2.61	0.0137	209	-23.76	0.64	0.07	25	14	92.02	10.28	3.80	24.25	639.33
08-12.4	70.50	0.0242	343	2.28	0.0098	139	-24.33	0.47	0.09	32	13	88.94	7.32	3.80	23.44	638.83
09-7.8	68.03	0.0226	332	5.16	0.0639	741	-17.23	1.93	0.22	73	163	83.39	6.15	3.34	24.98	633.9
13-45.5	86.20	0.0387	449	1.54	0.0639	741	-17.23	1.93	0.66	296	488	142.71	48.07	3.34	42.76	645
08-12.4	67.65	0.0247	365	2.15	0.0106	157	-25.03	0.5	0.09	34	15	91.01	7.98	3.34	27.27	638.83
08-5.1	68.82	0.0319	463	1.51	0.0240	349	-22.47	0.88	0.10	48	37	117.60	18.06	3.34	35.24	640
13-18.7	78.29	0.0437	559	2.54	0.0166	212	-21.66	0.44	0.67	373	142	161.27	12.49	3.34	48.32	646.42
08-20.4	60.34	0.0271	449	2.33	0.0414	686	-23.36	1.78	0.05	20	31	99.84	31.17	3.34	29.91	638
13-40.8	62.29	0.0285	458	1.64	0.0116	186	-20.69	0.47	0.65	300	122	105.08	8.71	3.34	31.48	645.29
09-7.1b	59.02	0.0263	446	5.47	0.0331	560	-21.80	1.47	0.14	61	76	97.06	24.89	3.34	29.08	634.1
09-3.6	84.78	0.0062	73	3.43	0.0265	313	-9.91	4.97	0.08	6	26	22.96	19.96	3.34	6.88	634.5

Continued on next page

Supplementary Table 8 – continued from previous page

Sample	Samp. mass (mg)	N (mg)	TN (ppm)	δ^{15}N	C (mg)	C (ppm)	δ^{13}C	at. C/N	Insoluble	WR N	WR C	N-peak	C-peak	N-blank	Peak/blank	Age
08-10.2b	62.37	0.0196	315	3.01	0.0156	251	-25.88	0.93	0.05	15	12	72.39	11.78	3.34	21.69	639
13-9.0	72.51	0.0405	558	2.75	0.0477	658	-17.00	1.38	0.31	174	205	149.15	35.90	3.34	44.69	647
08-9.1	54.24	0.0134	247	2.73	0.0127	233	-22.89	1.1	0.05	11	11	49.49	9.53	3.34	14.83	639.67
08-10.2c	73.98	0.0282	381	1.67	0.0131	177	-23.96	0.54	0.13	49	23	103.82	9.87	3.34	31.11	639.5
09-6.3	81.87	0.0044	54	2.87	0.0304	372	-5.32	8.01	0.07	4	25	16.33	22.90	3.34	4.89	634.3
09-0.1	64.27	0.0239	372	4.21	0.0245	382	-25.48	1.2	0.05	19	20	88.24	18.46	3.34	26.44	635
08-8.4	73.73	0.0294	398	2.10	0.0204	276	-25.27	0.81	0.08	33	23	108.03	15.34	3.30	32.70	639.83
09-7.8	79.24	0.0266	335	5.36	0.0081	102	-25.03	0.35	0.22	73	22	97.75	6.07	3.30	29.59	633.9
08-12.4	81.95	0.0291	356	2.13	0.0133	163	-25.20	0.53	0.09	34	15	107.20	10.04	3.30	32.45	638.83
08-5.1	90.53	0.0415	459	1.55	0.0311	343	-22.40	0.87	0.10	48	36	152.80	23.43	3.30	46.26	640
09-7.1b	73.20	0.0327	447	5.61	0.0415	567	-22.10	1.48	0.14	61	77	120.25	31.27	3.30	36.40	634.1
13-9.0	102.53	0.0562	548	2.62	0.0672	656	-17.10	1.4	0.31	171	205	206.61	50.67	3.30	62.55	647
13-34.8	100.89	0.0409	405	1.69	0.0644	638	-18.52	1.84	0.59	241	379	150.29	48.50	3.30	45.50	645.57
13-18.7	98.71	0.0496	503	2.22	0.0189	192	-20.99	0.44	0.67	336	128	182.57	14.25	3.30	55.27	646.42
13-24.8	95.24	0.0443	465	2.01	0.1714	1799	-16.18	4.51	0.45	207	801	162.89	129.13	3.30	49.31	646.18
09-1.6b	96.55	0.0244	253	4.60	0.0087	90	-21.21	0.42	0.26	67	24	90.41	6.59	2.79	32.40	634.8
09-1.6a	107.92	0.0084	78	3.74	0.0257	238	-15.83	3.56	0.10	8	24	31.17	19.43	2.79	11.17	634.7
09-0.6	90.49	0.0229	253	3.58	0.0083	92	-21.05	0.42	0.20	51	19	84.87	6.28	2.79	30.41	634.9
MR	10.78	0.0119	1100	5.55	0.8245	76464	-37.77	81.09		1100	76464	42.65	617.68	3.77	11.32	
MR	10.31	0.0117	1132	5.51	0.7876	76365	-37.72	78.69		1132	76365	42.95	590.01	3.80	11.32	
MR	11.06	0.0124	1124	5.56	0.8428	76183	-37.67	79.08		1124	76183	45.84	634.36	3.34	13.73	
MR	11.84	0.0136	1153	5.57	0.9037	76341	-37.70	77.25		1153	76341	50.21	681.03	3.30	15.20	
MR	10.67	0.0122	1139	5.19	0.8179	76637	-37.76	78.49		1139	76637	45.07	619.13	2.79	16.15	

Supplementary References

1. Scholten, S. The distribution of nitrogen isotopes in sediments. *Geologica Ultraiectina* **81,** 101 (1991).
2. Williams, L. B. *et al.* Nitrogen isotope geochemistry of organic matter and minerals during diagenesis and hydrocarbon migration. *Geochimica et Cosmochimica Acta* **59,** 765–779 (1995).
3. Thomazo, C. *et al.* Biological activity and the Earth's surface evolution: Insights from carbon, sulfur, nitrogen and iron stable isotopes in the rock record. *Comptes Rendus Palevol* **8,** 665–678 (2009).
4. Clauer, N & Kröner, A. Strontium and argon isotopic homogenization of pelitic sediments during low-grade regional metamorphism: the Pan-African upper Damara sequence of northern Namibia (South West Africa). *Earth and planetary science letters* **43,** 117–131 (1979).
5. Hoffman, P. F., Tirrul, R., King, J., St-Onge, M. & Lucas, S. *Axial projections and modes of crustal thickening, eastern Wopmay orogen* (eds Clark Jr., S., Burchfiel, B. & Suppe, J.) **pp. 1-29** (Geological Society of America - Special paper, 1988).
6. Bebout, G. & Fogel, M. Nitrogen-isotope compositions of metasedimentary rocks in the Catalina Schist, California: implications for metamorphic devolatilization history. *Geochimica et Cosmochimica Acta* **56,** 2839–2849 (1992).
7. Palya, A. P., Buick, I. S. & Bebout, G. E. Storage and mobility of nitrogen in the continental crust: Evidence from partially melted metasedimentary rocks, Mt. Stafford, Australia. *Chemical Geology* **281,** 211–226 (2011).
8. Ader, M. *et al.* Interpretation of the nitrogen isotopic composition of Precambrian sedimentary rocks: Assumptions and perspectives. *Chemical Geology* **429,** 93–110 (2016).
9. Liu, Y. *et al.* In situ analysis of major and trace elements of anhydrous minerals by LA-ICP-MS without applying an internal standard. *Chemical Geology* **257,** 34–43 (2008).
