# Supplementary material for: Marine oxygen production and open water supported an active nitrogen cycle during the Marinoan Snowball Earth
Source: Nat Commun. 2017 Nov 6;8:1316. doi: 10.1038/s41467-017-01453-z (PMC5673069; doi:10.1038/s41467-017-01453-z)
Supplement: Supplementary file 3 — Description of Additional Supplementary Files [file 41467_2017_1453_MOESM3_ESM.pdf]

Description of Additional Supplementary Files

File Name: Supplementary Data 1
Description: N and C analyses concentration and isotopic analyses, performed at the University of Washington Isolab

File Name: Supplementary Data 2
Description: Whole rock solution ICP-MS trace element analyses.

File Name: Supplementary Data 3
Description: Laser ablation ICP-MS analyses

File Name: Supplementary Data 4
Description: Fe-speciation data
